# Rapid Continuous Ruthenium-Catalysed Transfer Hydrogenation of Aromatic Nitriles to Primary Amines

**DOI:** 10.1055/s-0036-1589096

**Published:** 2017-08-21

**Authors:** Ricardo Labes, Davir González-Calderón, Claudio Battilocchio, Carlos Mateos, Graham R. Cumming, Oscar de Frutos, Juan A. Rincón, Steven V. Ley

**Affiliations:** a Innovative Technology Centre, Department of Chemistry, University of CambridgeLensfield Road, Cambridge, CB2 1EWUKsvl1000@cam.ac.uk; b Centro de Investigación Lilly S.A.Avda. de la Industria 30, Alcobendas-Madrid 28108Spainc.mateos@lilly.com

**Keywords:** nitrile reduction, transfer hydrogenation, ruthenium, primary amine, continuous flow

## Abstract

A continuous flow method for the selective reduction of aromatic nitriles to the corresponding amine is reported. The method is based on a ruthenium-catalysed transfer-hydrogenation process, requires no additives, and uses isopropanol as both solvent and reducing agent. The process utilizes 1 mol% of the commercially available [Ru( *p* -cymene)Cl _2_ ] _2_ , with a residence time of ca. 9 min, and a throughput of 50 mmol/h. The method was successfully applied to a range of aromatic nitriles providing the corresponding primary amines in good yields.

Amines are key intermediates in the production of fine chemicals, active pharmaceutical ingredients (APIs), agro-chemicals, as well as many natural products. 1 In particular, primary amines are important synthetic building blocks, as these are used in many processes such as the Buchwald–Hartwig coupling reactions, 2 hydroaminations, 3 and alcohol amination by hydrogen-borrowing strategies 4 5 6 7 for example.

The reduction of nitriles is among the most common route to generate the corresponding primary amines. 8 The use of hydrides in stoichiometric amounts such as LiAlH _4_ is effective for this transformation, but catalytic hydrogenation methods using Raney Nickel, for example, are often preferred, however, these are not without problems. Despite being known for decades, 9 transfer-hydrogenation processes (TH) have gained in interest recently, 10 also with applications to nitrile reduction. 11 12 13 14 Transfer-hydrogenation techniques are attractive as they eliminate the need for pressurized hydrogen gas typical of many catalytic methods. Both direct hydrogenation (employing H _2_ ), and TH methodologies can, however, suffer from selectivity issues and lead to several byproducts being formed in these reactions (Scheme 1 ). Several recent attempts have been reported to solve these selectivity problems employing catalysts and H _2_ gas 8 and selective TH reactions. 11 12 13


**Scheme 1** Side reactions for the transfer hydrogenation and hydrogenation of nitriles


To the best of our knowledge, while hydrogenation reactions have been thoroughly investigated in continuous flow, 15 16 17 18 19 20 including nitrile reductions to the correspondent primary amine 21 and direct reductive amination, 22 no continuous transfer hydrogenation of nitriles to primary amines has been reported so far. However, we could expect to see distinct advantages from the approach, especially in terms of safety and selectivity.

Beller and coworkers have reported the use of 2-butanol in the transfer hydrogenation of nitriles to obtain the corresponding primary amine while simultaneously reducing the formation of byproducts. 11 The same group also reported a heterogeneous TH using Pd/C and ammonium formate as a hydrogen source 12 and the use of 2-propanol with NaOH to afford the alkylated secondary amines. 23 On the other hand Zhou and Liu assessed two cobalt catalysts for TH of nitriles that led to the selective preparation of primary, secondary, or tertiary products. Here the solvent choice played a crucial role on the outcome of the reaction. 13

Notably, three recent reports using transfer hydrogenation to reduce nitriles with a ruthenium catalyst led to the *N* -isopropylidene derivatives ( **4** , Scheme 1 ) as the main product through a further coupling and reduction. These studies use KO *^t^* Bu as base and relatively long reaction times. 14 24 25 Recently, the use of a nickel catalyst and 1,4-butanediol as hydrogen source to afford *N* -benzylidenes as main products of the transfer hydrogenation nitrile reduction have been observed. 26

Even with all these advances, we felt there was a need for a practical, safe, and selective method for the transfer hydrogenation of nitriles, tolerant to a wide aromatic functionality. Inspired by our recent studies towards the chemoselective continuous ruthenium-catalysed hydrogen-transfer oxidation of secondary alcohols, 27 we became interested in expanding the application scope of the system. Among other advantages of using a continuous-flow system is the precise control of residence times and heating regimes.

By analogy with our previous approach to continuous transfer hydrogenation, we devised a simple system comprising of a pump, a heated coil, and a backpressure regulator (BPR). Initial screening was performed using a Uniqsis FlowSyn 28 unit equipped with a 20 mL stainless-steel coil reactor operating at the temperature indicated (Table 1 ).

**Table:** **Table 1** Initial Optimisation for the Continuous TH of Nitriles

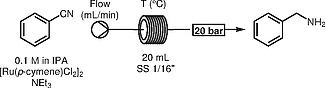					
Entry	Residence time (min)	Temp (°C)	Cat. (mol%)	Et _3_ N (equiv)	Yield (%) ^a^
1	40	100	0	2	0
2	40	100	1	2	50
3	40	125	1	2	75
4	40	150	1	2	85
5	40	150	1	0	85
6 ^b^	40	150	1	2	0
7	40	150	0.5	0	80
8	40	150	5	0	66
9	20	150	1	0	85
10	10	150	1	0	85

^a^ Yield of primary amine by ^1^ H NMR analysis (1,4-dinitrobenzene as internal standard) of the crude mixture after solvent removal.

^b^ Using (Ph _3_ P) _3_ RuCl _2_ as catalyst.

Our study began employing triethylamine as a base, as it proved to be beneficial in our previous work employing the same catalyst. 27 It was quickly realised that triethylamine was not in fact necessary for this transformation to proceed (Table 1 ).

Interestingly, Beller’s report suggests that no conversion was observed using the same catalyst [Ru( *p* -cymene)Cl _2_ ] _2_ and 2-butanol at 120 °C for ten minutes in batch. 11 We were pleasantly surprised to find that under the conditions described, even at 100 °C (Table 1 , entry 2), the expected primary amine could be observed in flow. By increasing the temperature to 150 °C the yield increased to 85% in the absence of phosphine ligands or base (Table 1 , entry 5). Concentration of the substrate in IPA was crucial to the outcome of the reaction. By increasing either the concentration of substrate or the amount of ruthenium catalyst, the amount of alkylated byproduct **5** increased. Residence time could be reduced to ten minutes maintaining the product yield. By reducing the residence time we diminished the amount of alkylated byproduct **5** . However, we could now observe some unreacted imine **2** , while the overall yield of primary amine was unchanged.

The reaction system was then transferred to a Phoenix reactor platform, 29 equipped with a 1/8′′ 35 mL stainless steel coil. This gave us the opportunity to work at even higher temperatures and pressures.

By precisely controlling the residence time of the solution we could halt the reaction at a specific equilibrium point between **1** and **5** (Scheme 1 ). The residence time was then optimized using benzonitrile as a model substrate. To enable a suitable downstream product isolation, we opted to isolate the primary amines as their hydrochloride salts, which could be easily separated by filtration, also enabling removal of any ruthenium residues. The general method was then applied to different nitriles (Table 2 ). 30

Notably, aromatic nitriles bearing ether, thioether, chloride, and amide functionalities were all successfully reduced in good yields and good degree of selectivity. A pyridine derivative (Table 2 , entry 16) was also converted into the desired primary amine in good yield. A nitrile compound containing an amide group (Table 2 , entry 15) selectively afforded the corresponding primary amine, however, substrates bearing more reactive carbonyl groups as in ­ketones or aldehydes led to a mixture of products.

Attempts to reduce aliphatic nitriles also led to poor yields due to reduced reactivity of these substrates. 31 This expected lower reactivity has been observed by others. 12 14 To further evaluate the robustness of the methodology, the system was operated continuously for two hours and afforded 4 g of benzylamine (Scheme 2 ).

**Table:** **Table 2** Substrate Scope for the Continuous TH of Nitriles

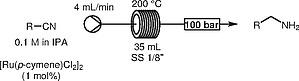		
Entry	Substrate	Yield (%) ^a^
1	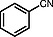	78
2	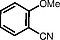	71 ^b^
3	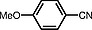	80
4	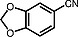	69
5	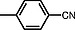	76
6	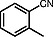	71
7	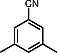	69
8	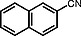	70
9	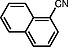	72
10	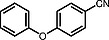	82
11	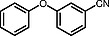	70
12	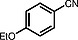	78
13	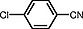	72
14	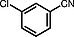	63
15	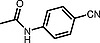	77
16	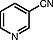	71 ^b^
17	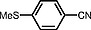	70

^a^ Isolated yield as hydrochloride salts.

^b^ Yield of primary amine by ^1^ H NMR analysis (1,4-dinitrobenzene as internal standard).


**Scheme 2** Transfer hydrogenation of nitriles, 2 h continuous operation


To conclude, we have developed a fast, continuous, ruthenium-catalysed transfer hydrogenation of aromatic nitriles to afford primary amines. The system uses a commercial ruthenium catalyst and IPA as solvent and hydrogen donor. This system was successfully applied to reduce 17 different nitriles affording their correspondent primary amines in good yields.
