# Supplementary material for: Rapid Continuous Ruthenium-Catalysed Transfer Hydrogenation of Aromatic Nitriles to Primary Amines
Source: Synlett. 2017 Aug 21;28(20):2855–8. doi: 10.1055/s-0036-1589096 (PMC6193226; doi:10.1055/s-0036-1589096)
Supplement: Supplementary file 1 — Supporting Information [file sup_st-2017-r0508-l_10-1055_s-0036-1589096.pdf]

Synlett
Accounts and Rapid Communications in Chemical Synthesis

Supporting Information
for DOI: 10.1055/s-0036-1589096
© Georg Thieme Verlag KG Stuttgart · New York 2017

Thieme

Supporting Information

Rapid Continuous Ru-Catalyzed Transfer Hydrogenation of Aromatic Nitriles to Primary Amines

Ricardo Labes,[†] *Davir G. Calderón,*[†] *Claudio Battilocchio,*[†] *Carlos Mateos,*[‡*] *Graham R. Cumming,*[‡] *Oscar de Frutos,*[‡] *Juan A. Rincón,*[‡] *Steven V. Ley.*[†*]

[†] Innovative Technology Centre, Department of Chemistry, University of Cambridge, Lensfield Road, Cambridge, CB2 1EW, U.K.

[‡] Centro de Investigación Lilly S.A., Avda. de la Industria 30, Alcobendas-Madrid 28108, Spain.

General experimental section

^{1}H-NMR spectra were recorded on a Bruker Avance DPX-600 spectrometer with the residual solvent peak as the internal reference (CD_3OD = 3.31 ppm). ^{1}H resonances are reported to the nearest 0.01 ppm. ^{13}C-NMR spectra were recorded on the same spectrometer with the central resonance of the solvent peak as the internal reference (CD_3OD = 49.00). All ^{13}C resonances are reported to the nearest 0.1 ppm. The multiplicity of ^{1}H signals are indicated as: s = singlet, d = doublet, dd = doublet of doublet, ddd = doublet of doublet of doublet, t = triplet, q = quadruplet, quint = quintet, sext = sextet, m = multiplet, br. = broad, or combinations of thereof. Coupling constants (*J*) are quoted in Hz and reported to the nearest 0.1 Hz. Where appropriate, averages of the signals from peaks displaying multiplicity were used to calculate the value of the coupling constant.

Unless stated otherwise, reagents were obtained from commercial sources and used without purification. 2-propanol HPLC grade was purchased from Fisher Scientific and degassed using a stream of argon prior to use. The removal of solvent under reduced pressure was carried out on a standard rotary evaporator.

Flow equipment

The system used in the continuous transfer hydrogenation of aromatic nitriles was composed by a HPLC pump (Series III), a ThalesNano Phoenix[1] reactor and back pressure regulator (Figure S1).

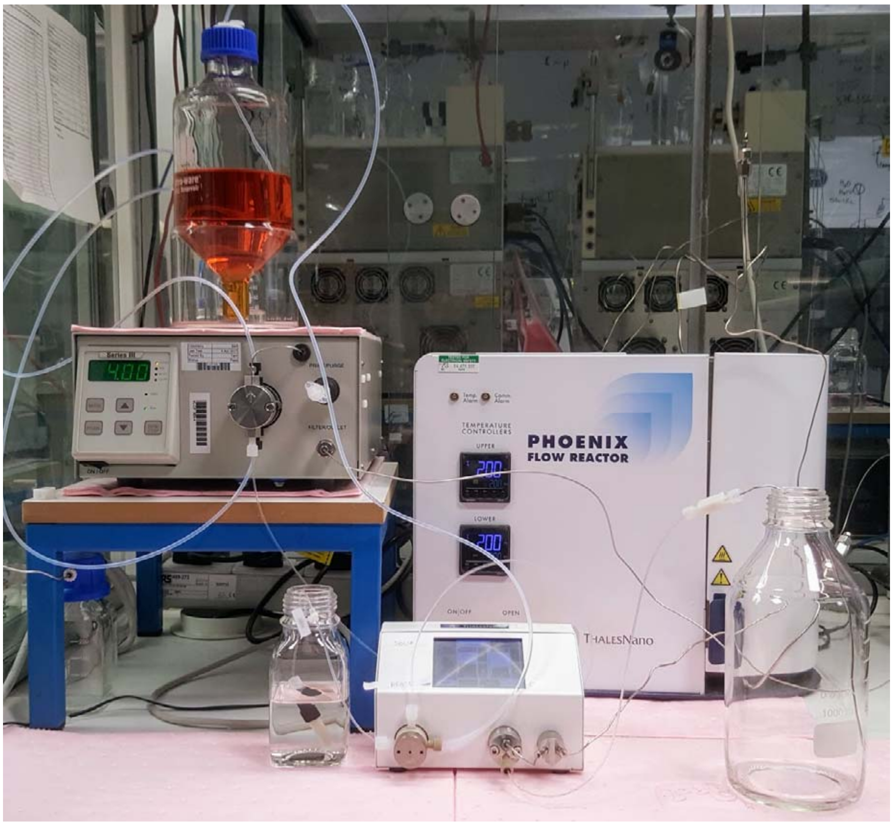

Figure S1: System composed by Series III HPLC pump, Phoenix reaction and back pressure regulator.

General method for the continuous transfer hydrogenation of nitriles

A solution (50 mL) containing the nitrile (5 mmol), and dichloro(*p*-cymene)ruthenium(II) dimer (0.05 mmol) in 2-propanol (solution was sonicated until the catalyst was solubilized 10-50 min) was pumped at 4 mL/min through the reactor coil heated at 200 °C. The Phoenix backpressure regulator was set to manual at 30%, which correlated to approximately 100 bar. A 10 mL fraction of the solution obtained from the system in steady state was used to prepare the hydrochloric salt by method A or B depending on substrate. Yields are reported as isolated hydrochloride salts, unless otherwise stated.

<u>Method A</u>: The collected fraction was concentrated under reduced pressure to approximately 5 mL, then HCl (4 mL, 1 M in dioxane) was added to the fraction and time allowed for solid formation. The solid was filtered, washed with hexane (30 mL) and diethylether (30 mL) before solvents and residual water were removed *in vacuo*.

Method B: HCl (4 mL, 1 M in dioxane), then hexane (4 mL) were added to the fraction and time allowed for solid formation. The solid was filtered, washed with hexane (30 mL) and diethylether (30 mL) before solvents and residual water were removed *in vacuo*.

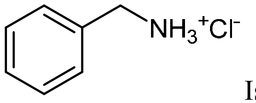

Isolated as a white solid using method B. The data for this compound is in agreement with that reported.[2]

^{1}H NMR (600 MHz, MeOD) δ 7.35 – 7.52 (m, 5H), 4.12 (s, 2H).

^{13}C NMR (151 MHz, MeOD) δ 133.01, 128.80, 128.79, 128.58, 42.96.

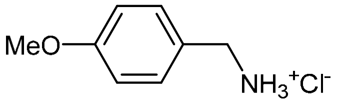

Isolated as a white solid using method B. The data for this compound is in agreement with that reported.[2]

^{1}H NMR (600 MHz, MeOD) δ 7.40 (d, J = 8.6 Hz, 2H), 7.00 (d, J = 8.6 Hz, 2H), 4.14 (s, 2H), 3.82 (s, 3H).

^{13}C NMR (151 MHz, MeOD) δ 160.75, 131.13, 122.75, 114.15, 54.40, 49.94.

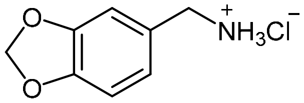

Isolated as a white solid using method A. The data for this compound is in agreement with that reported.[3]

^{1}H NMR (600 MHz, MeOD) δ 7.02 – 6.94 (m, 2H), 6.90 (m, 1H), 6.02 (s, 2H), 4.13 (s, 2H).

^{13}C NMR (151 MHz, MeOD) δ 148.81, 148.35, 124.43, 123.86, 109.54, 108.27, 101.63, 50.34.

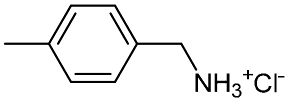

Isolated as a white solid using method A. The data for this compound is in agreement with that reported.[4]

^{1}H NMR (600 MHz, MeOD) δ 7.37 (d, J = 8.0 Hz, 2H), 7.29 (d, J = 8.0 Hz, 2H), 4.18 (s, 2H), 2.38 (s, 3H).

^{13}C NMR (151 MHz, MeOD) δ 139.56, 129.62, 129.48, 127.97, 50.26, 19.84.

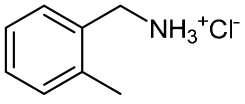

Isolated as a white solid using method B. The data for this compound is in agreement with that reported.[5]

^{1}H NMR (600 MHz, MeOD) δ 7.45 (d, *J* = 8.1 Hz, 1H), 7.26 – 7.39 (m, 3H), 4.33 (s, 2H), 2.37 (s, 3H).

^{13}C NMR (151 MHz, MeOD) δ 137.56, 130.84, 130.05, 129.54, 129.29, 126.45, 47.25, 17.75.

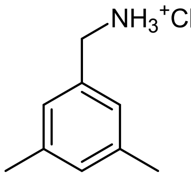

Isolated as a white solid using method A. The data for this compound is in agreement with that reported.[6]

^{1}H NMR (600 MHz, MeOD) δ 7.09 (d, J = 14.4 Hz, 3H), 4.12 (s, 2H), 2.34 (s, 6H).

^{13}C NMR (151 MHz, MeOD) δ 138.80, 130.71, 130.66, 127.21, 50.46, 19.80.

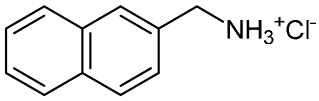

Isolated as a white solid using method A. The data for this compound is in agreement with that reported.[5]

^{1}H NMR (600 MHz, MeOD) δ 8.09 – 7.84 (m, 4H), 7.66 – 7.48 (m, 3H), 4.47 (s, 2H).

^{13}C NMR (151 MHz, MeOD) δ 133.61, 133.25, 129.53, 128.81, 128.31, 127.71, 127.42, 126.85, 126.55, 126.16, 50.92.

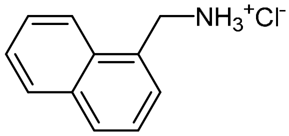

Isolated as a white solid using method B. The data for this compound is in agreement with that reported.[2]

^{1}H NMR (600 MHz, $CDCl_3$) δ 8.04 (d, J = 8.1 Hz, 2H), 8.00 (d, J = 7.5 Hz, 1H), 7.73 (d, J = 7.0 Hz, 1H), 7.61 (m, 3H), 4.87 (s, 2H).

^{13}C NMR (151 MHz, $CDCl_3$) δ 137.99, 135.33, 134.31, 133.25, 132.75, 131.06, 130.78, 130.18, 129.06, 126.20, 51.91.

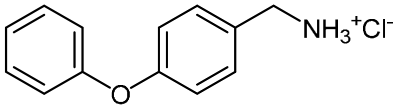

Isolated as colorless crystals using method A. The data for this compound is in agreement with that reported.[5]

^{1}H NMR (600 MHz, MeOD) δ 7.47 (d, J = 8.6 Hz, 2H), 7.39 (t, J = 8.2 Hz, 2H), 7.17 (t, J = 7.4 Hz, 1H), 7.03 (dd, J = 14.3, 8.2 Hz, 4H), 4.22 (s, 2H).

^{13}C NMR (151 MHz, MeOD) δ 158.86, 156.29, 131.47, 129.66, 125.33, 123.80, 119.13, 118.29, 50.00.

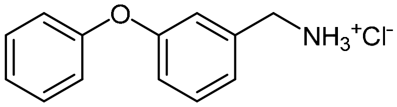

Isolated as a white solid using method B.

^{1}H NMR (600 MHz, MeOD) δ 7.45 (t, J = 8.0 Hz, 1H), 7.40 (t, J = 8.0 Hz, 2H), 7.21 (d, J = 7.6 Hz, 1H), 7.18 (t, J = 7.4 Hz, 1H), 7.14 (s, 1H), 7.07 (dd, J = 8.2, 2.3 Hz, 1H), 7.04 (d, J = 7.8 Hz, 2H), 4.21 (s, 2H).

^{13}C NMR (151 MHz, MeOD) δ 158.21, 156.52, 132.89, 130.42, 129.70, 124.13, 123.67, 119.30, 119.18, 118.97, 50.37.

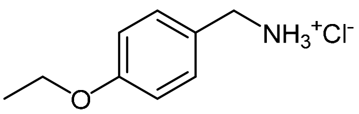

Isolated as a white solid using method A.

^{1}H NMR (600 MHz, MeOD) δ 7.38 (d, J = 8.7 Hz, 2H), 6.98 (d, J = 8.7 Hz, 2H), 4.13 (s, 2H), 4.06 (q, J = 7.0 Hz, 2H), 1.39 (t, J = 7.0 Hz, 3H).

^{13}C NMR (151 MHz, MeOD) δ 160.04, 131.08, 122.62, 114.66, 63.22, 49.97, 13.60.

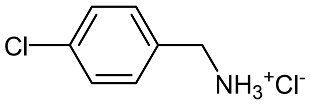

Isolated as a white solid using method B. The data for this compound is in agreement with that reported.[2]

^{1}H NMR (600 MHz, MeOD) δ 7.35-7.41 (m, 4H), 4.25 (s, 2H).

^{13}C NMR (151 MHz, MeOD) δ 135.43, 131.37, 129.67, 129.00, 50.00.

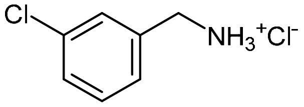

Isolated as a white solid using method B. The data for this compound is in agreement with that reported.[5]

^{1}H NMR (600 MHz, MeOD) δ 7.59 (s, 1H), 7.53 – 7.43 (m, 3H), 4.29 (s, 2H).

^{13}C NMR (151 MHz, MeOD) δ 134.63, 133.08, 130.46, 129.74, 129.47, 128.08, 50.22.

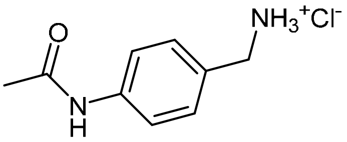

Isolated as a white solid using method A. The data for this compound is in agreement with that reported.[2]

^{1}H NMR (600 MHz, MeOD) δ 7.66 (d, J = 8.6 Hz, 2H), 7.44 (d, J = 8.6 Hz, 2H), 4.19 (s, 2H), 2.14 (s, 3H).

^{13}C NMR (151 MHz, MeOD) δ 170.43, 139.86, 130.30, 126.13, 119.96, 50.10, 22.43.

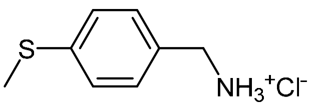

Isolated as a white solid using method A. The data for this compound is in agreement with that reported.[2]

^{1}H NMR (600 MHz, MeOD) δ 7.41 (d, J = 8.3 Hz, 2H), 7.34 (d, J = 8.3 Hz, 2H), 4.19 (s, 2H), 2.51 (s, 3H).

^{13}C NMR (151 MHz, MeOD) δ 141.29, 130.14, 127.39, 125.92, 50.16, 13.59.

^{1}H NMR for isolated compounds

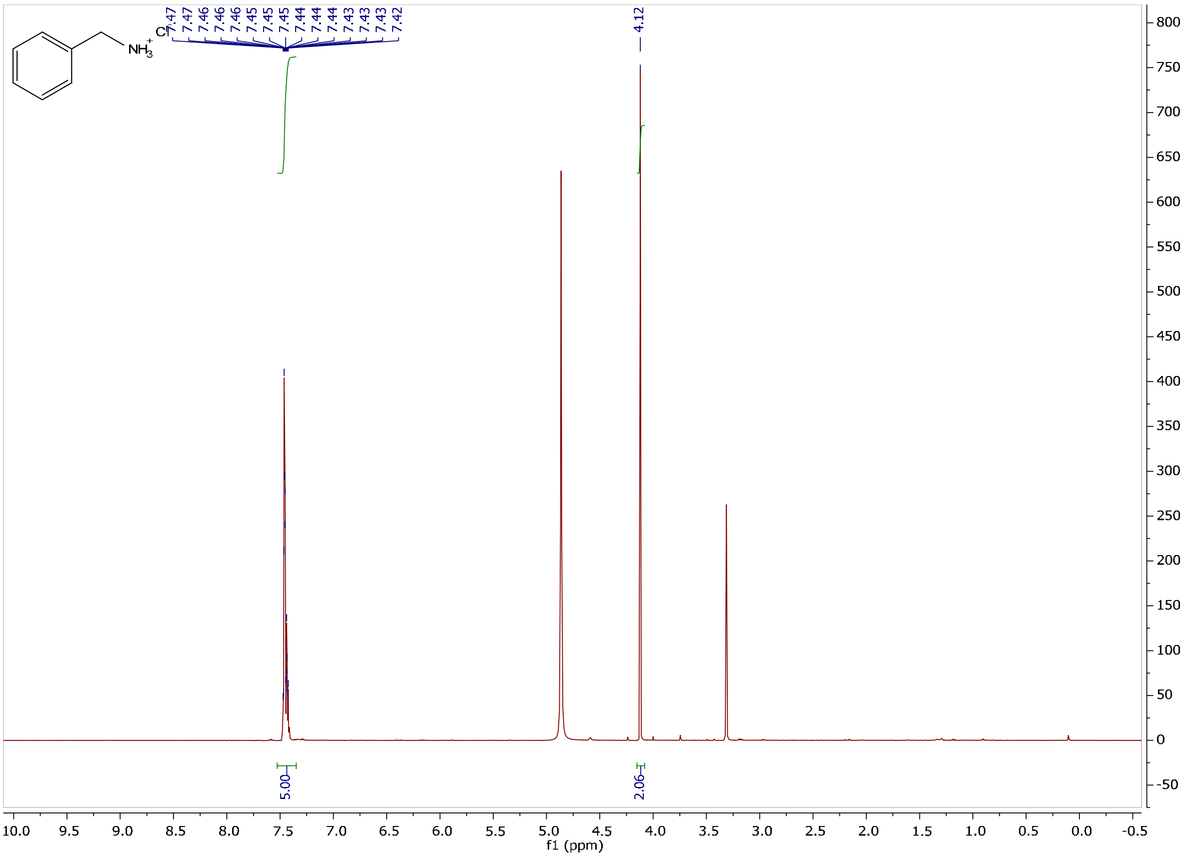

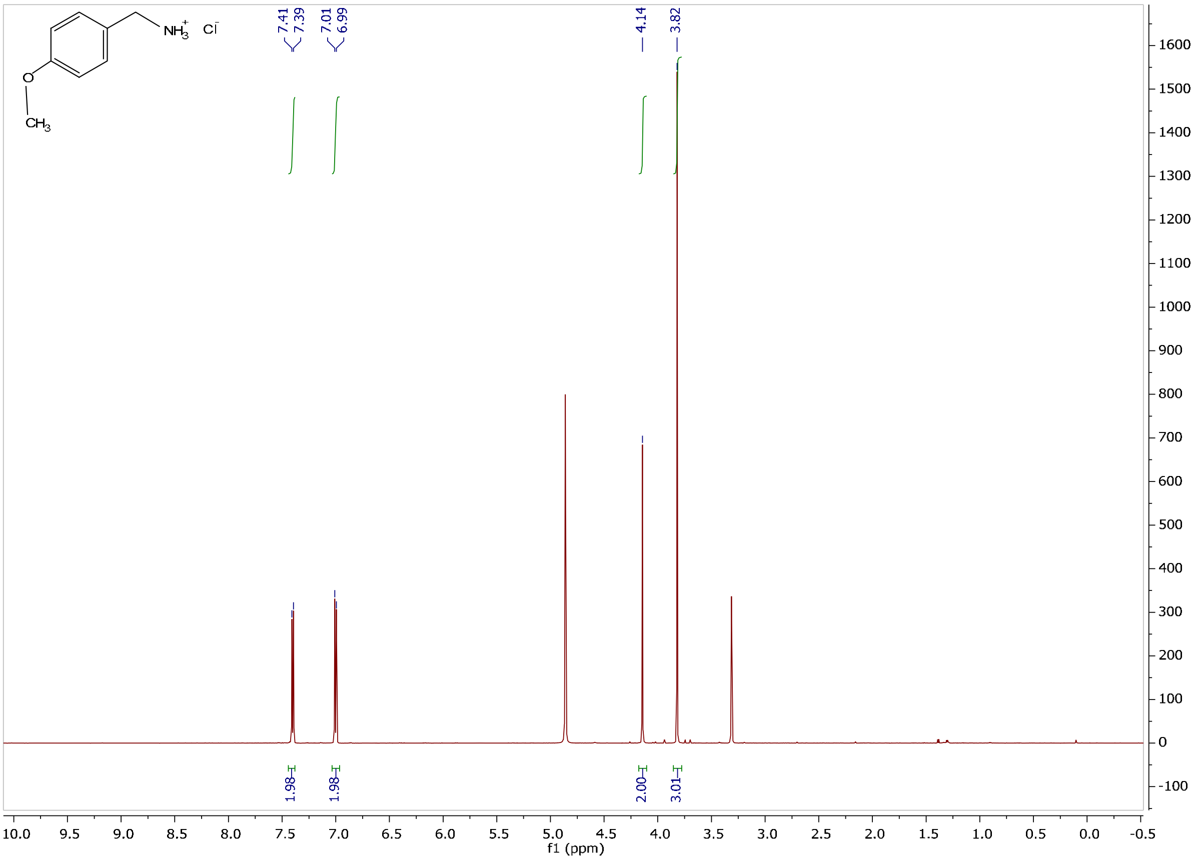

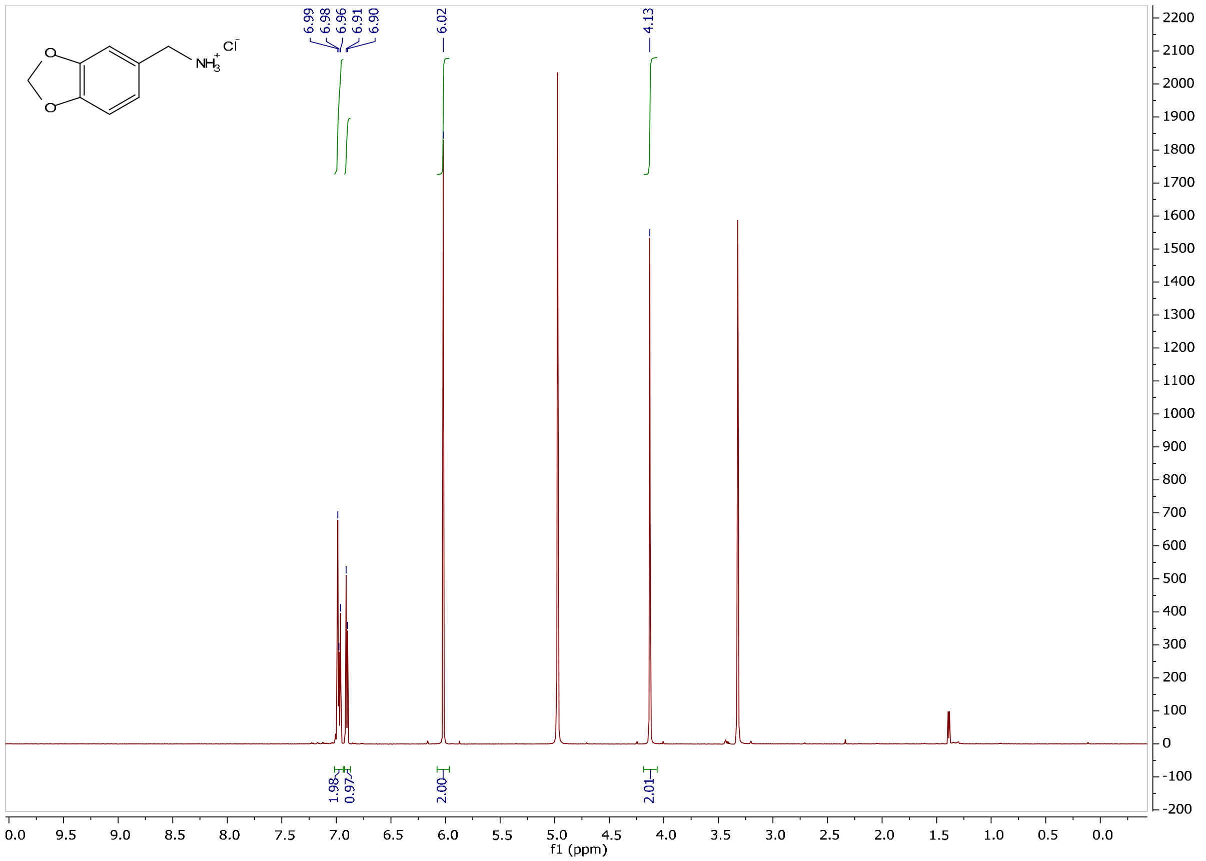

Cl⁻
NH₃⁺
6.99
6.98
6.96
6.91
6.90
6.02
4.13
1.98
0.97
2.00
2.01
f1 (ppm)

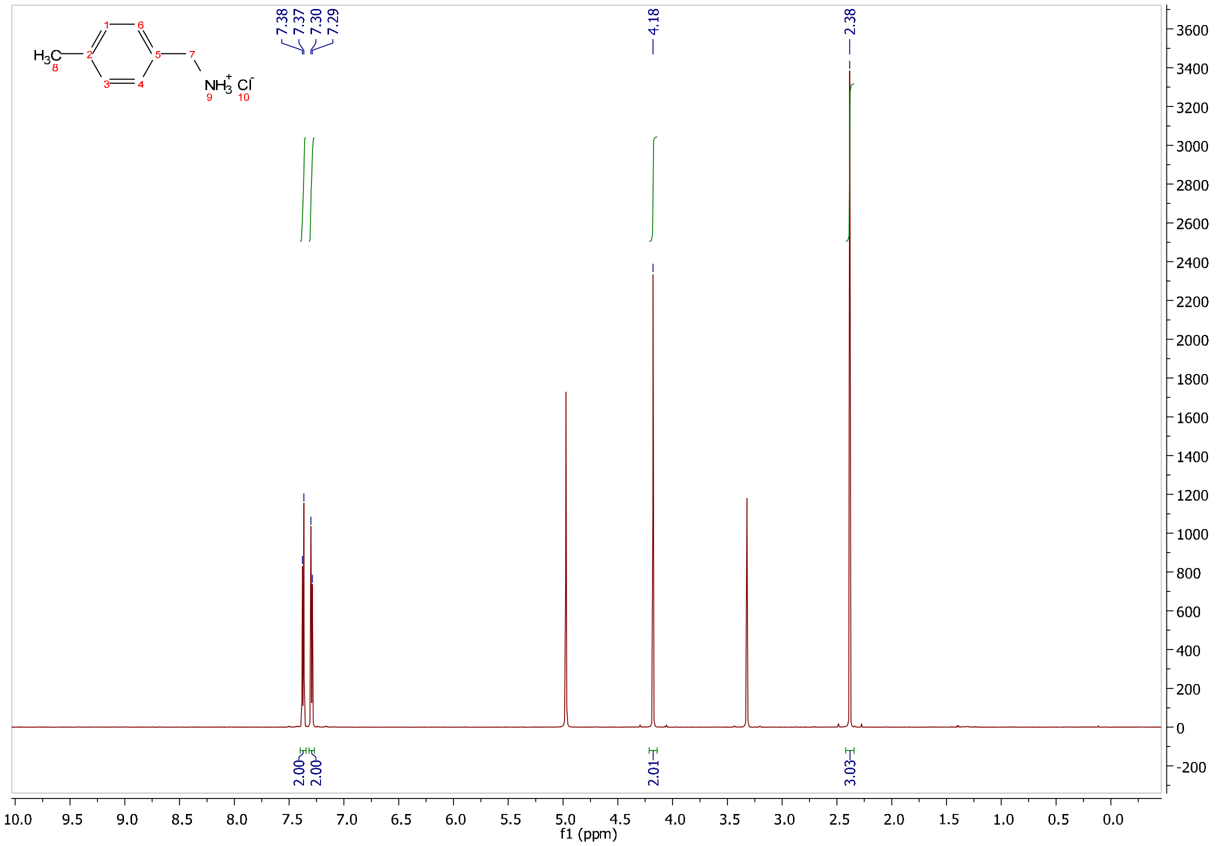

H₃C
NH₃⁺ Cl⁻
7.38
7.37
7.30
7.29
4.18
2.38
2.00
2.00
2.01
3.03
f1 (ppm)

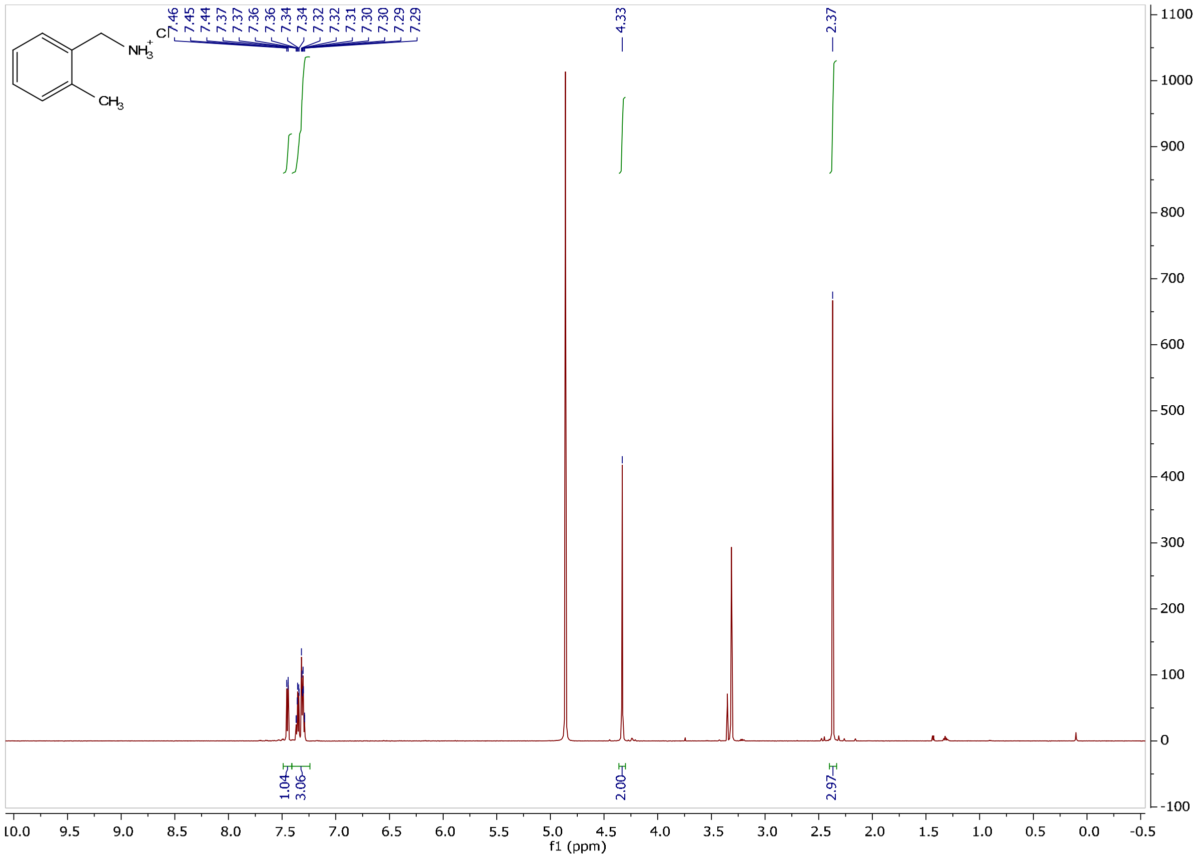
NH3+ Cl-
CH3
7.46
7.45
7.44
7.37
7.37
7.36
7.36
7.34
7.34
7.32
7.32
7.31
7.30
7.30
7.29
7.29
4.33
2.37
1.04
3.06
2.00
2.97
f1 (ppm)

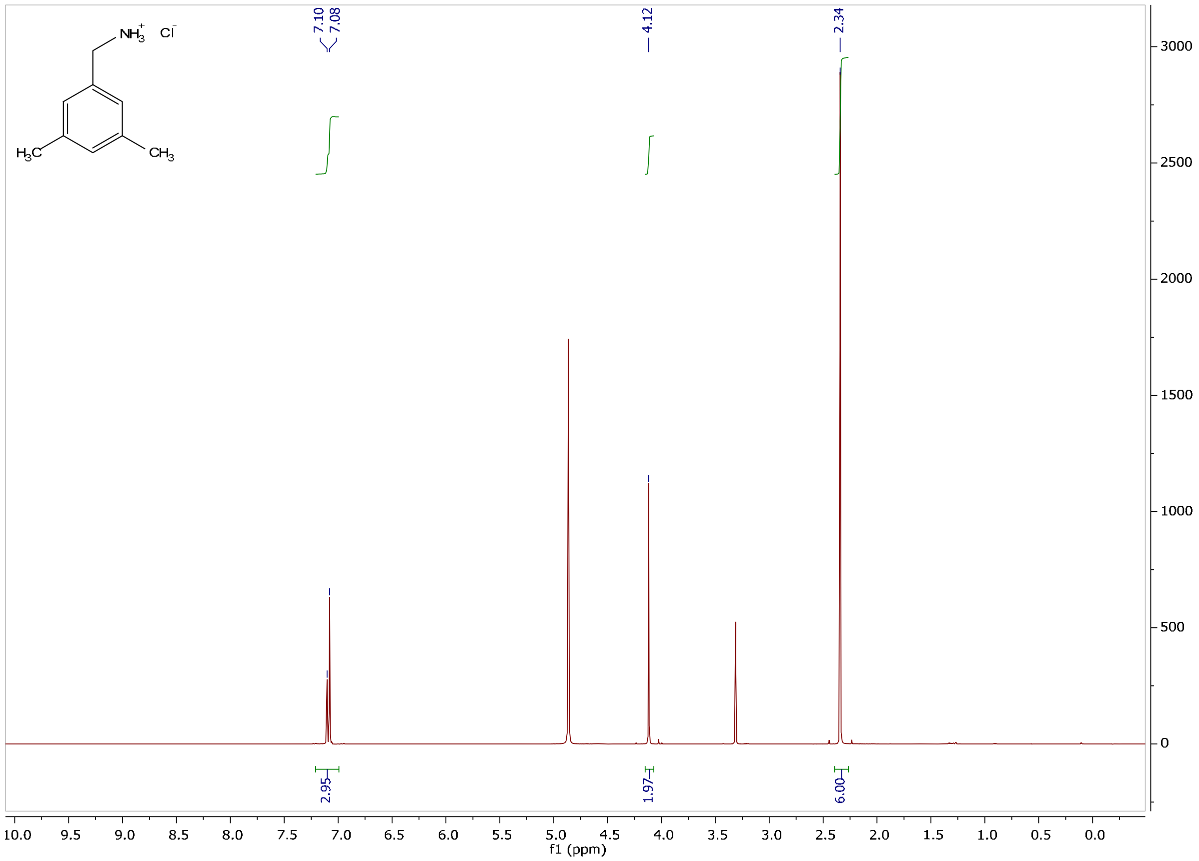
NH3+ Cl-
H3C
CH3
7.10
7.08
4.12
2.34
2.95
1.97
6.00
f1 (ppm)

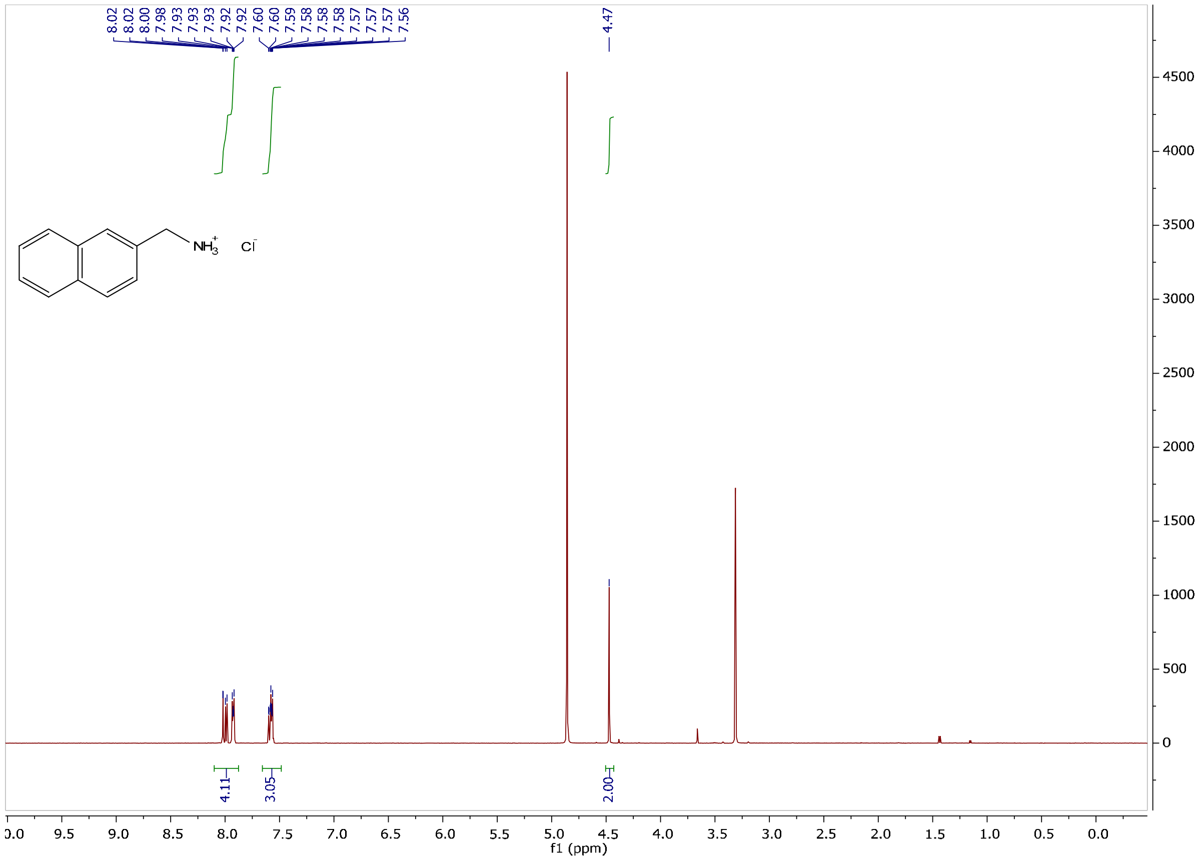

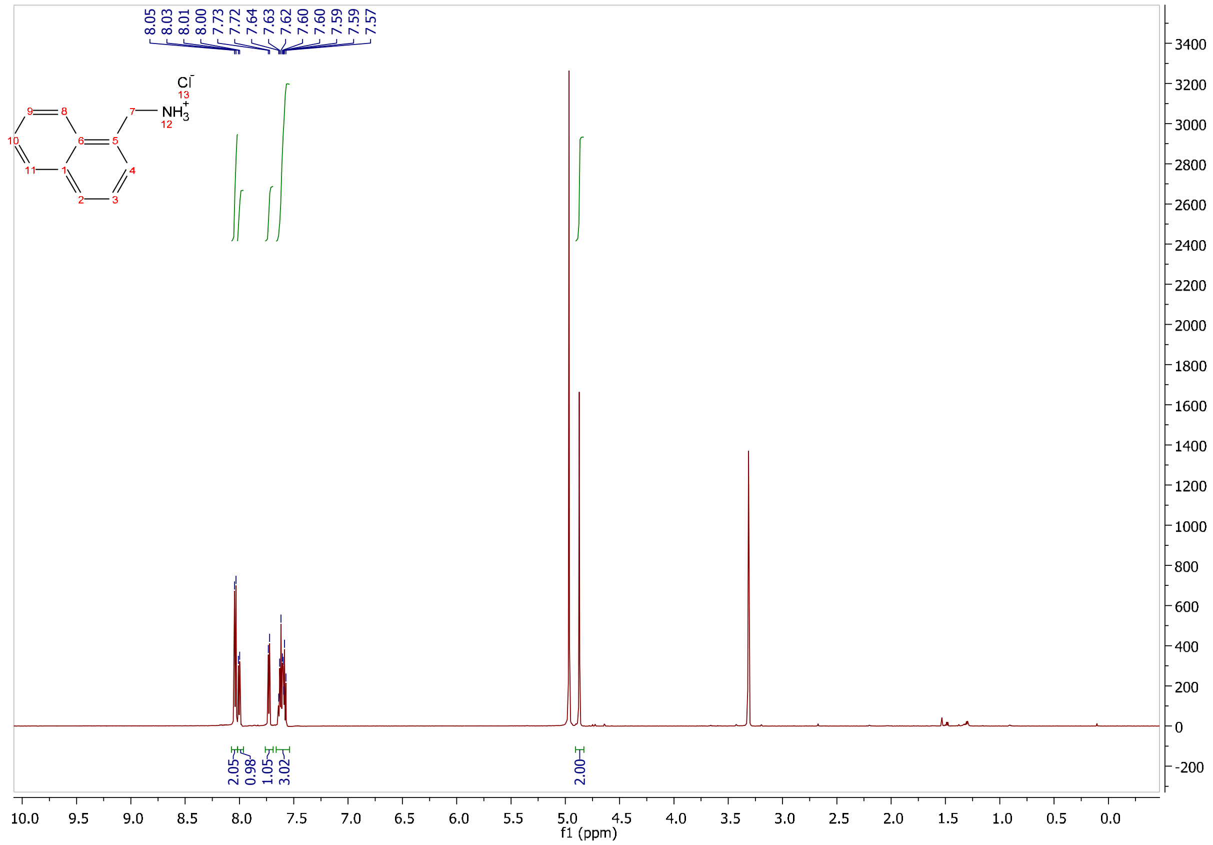

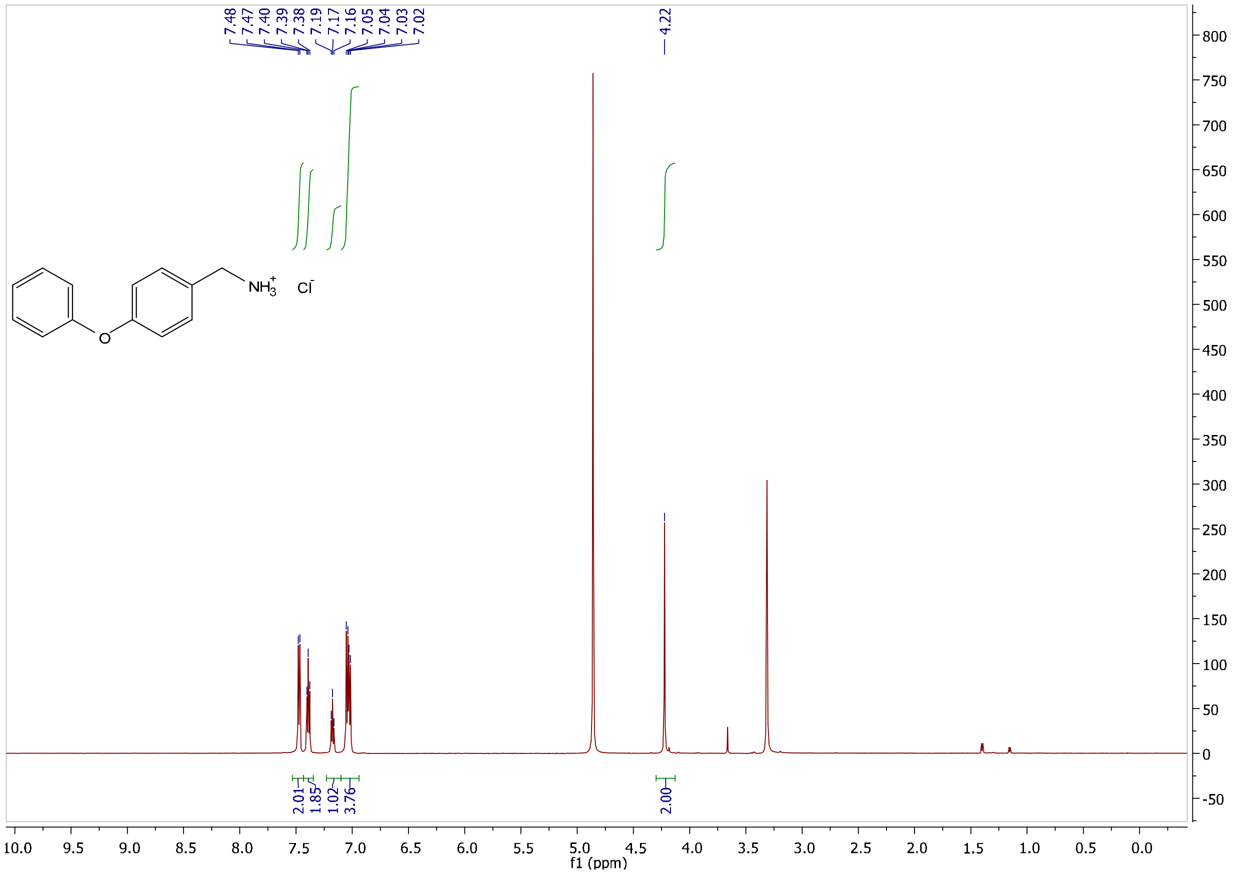
7.48
7.47
7.40
7.39
7.38
7.19
7.17
7.16
7.05
7.04
7.03
7.02
4.22
NH3+ Cl-
O
2.01
1.85
1.02
3.76
2.00
f1 (ppm)

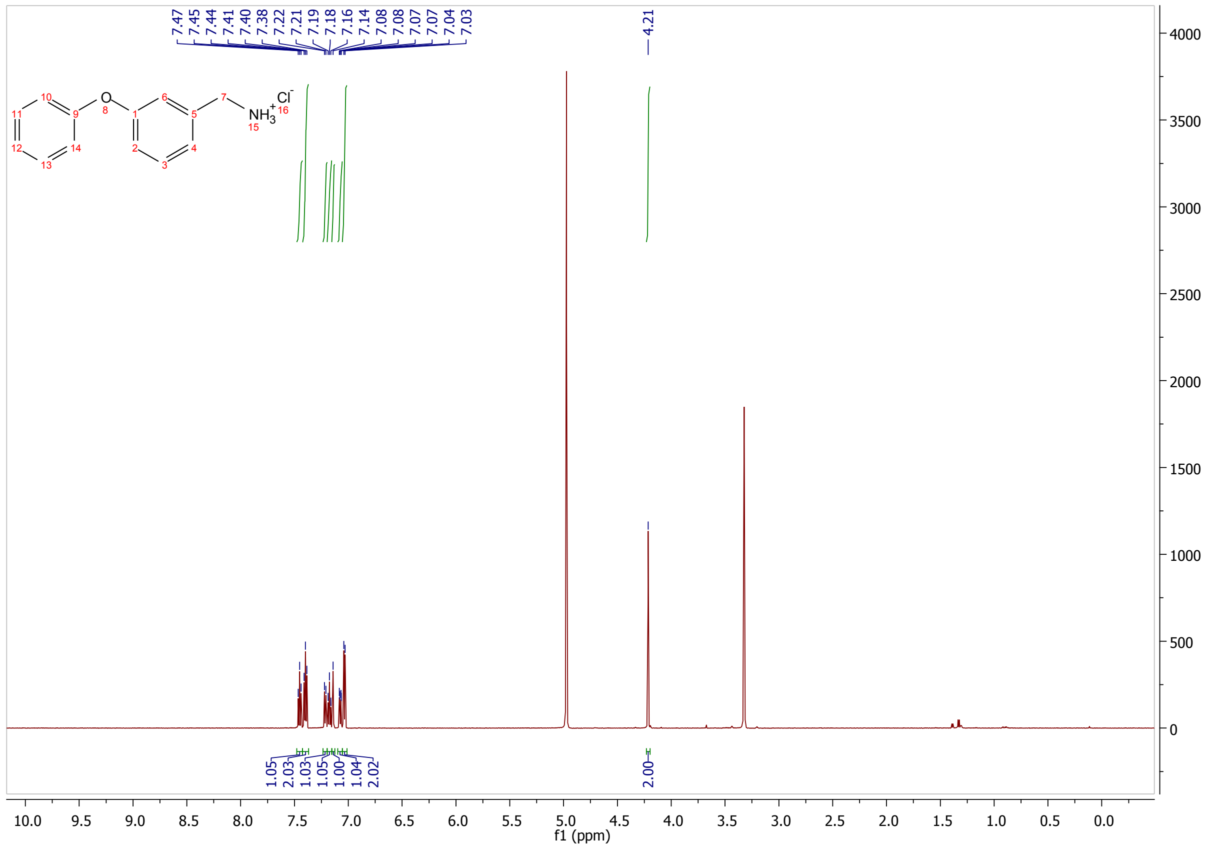
7.47
7.45
7.44
7.41
7.40
7.38
7.22
7.21
7.19
7.18
7.16
7.14
7.08
7.08
7.07
7.07
7.04
7.03
4.21
NH3+ Cl-
O
1.05
2.03
1.03
1.05
1.00
1.04
2.02
2.00
f1 (ppm)

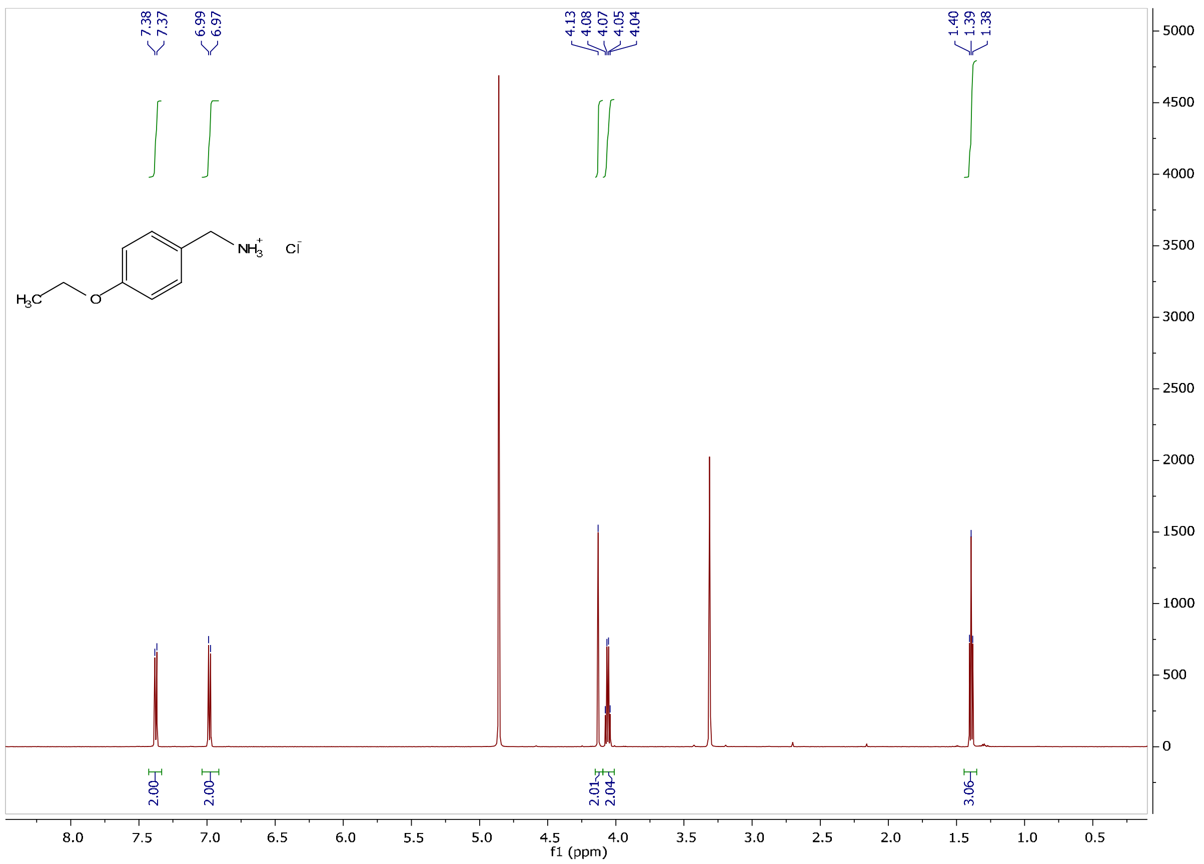
7.38
7.37
6.99
6.97
4.13
4.08
4.07
4.05
4.04
1.40
1.39
1.38
H3C
O
NH3+
Cl−
2.00
2.00
2.01
2.04
3.06
f1 (ppm)

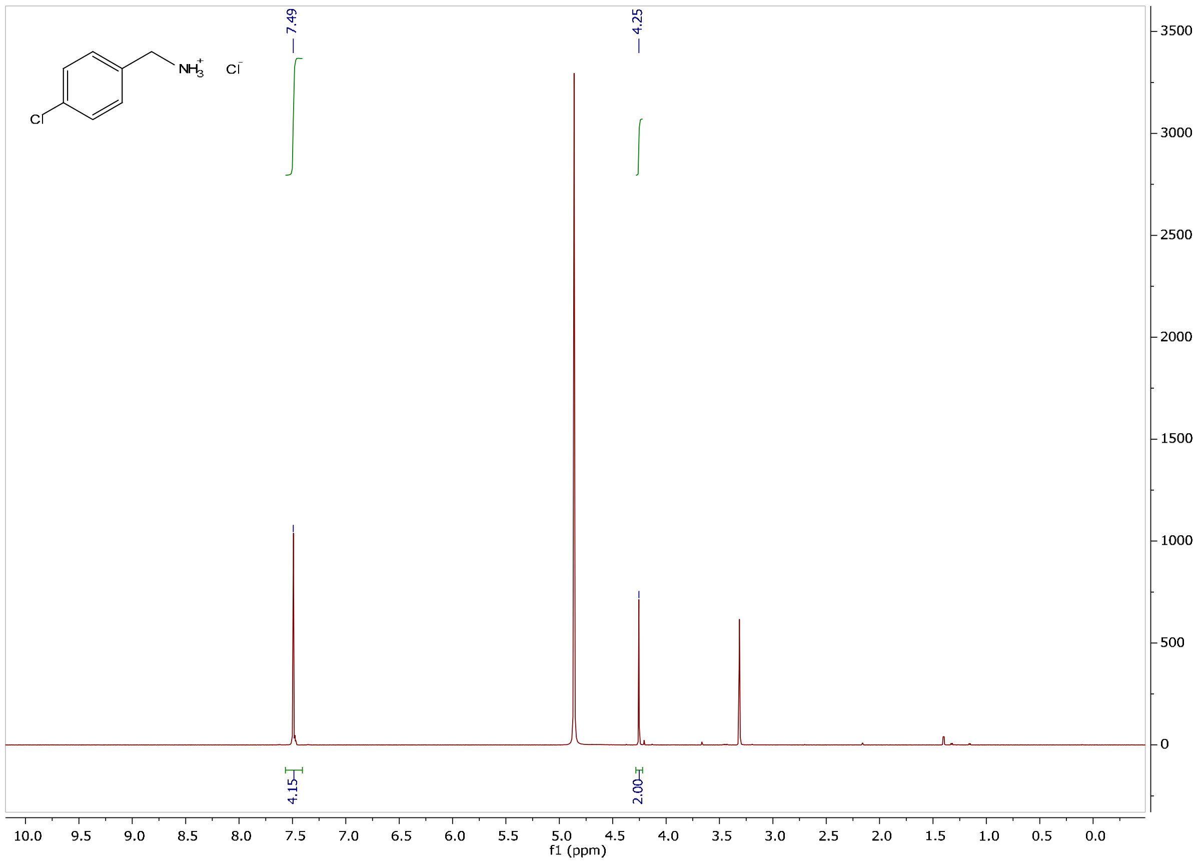
7.49
4.25
Cl
NH3+
Cl−
4.15
2.00
f1 (ppm)

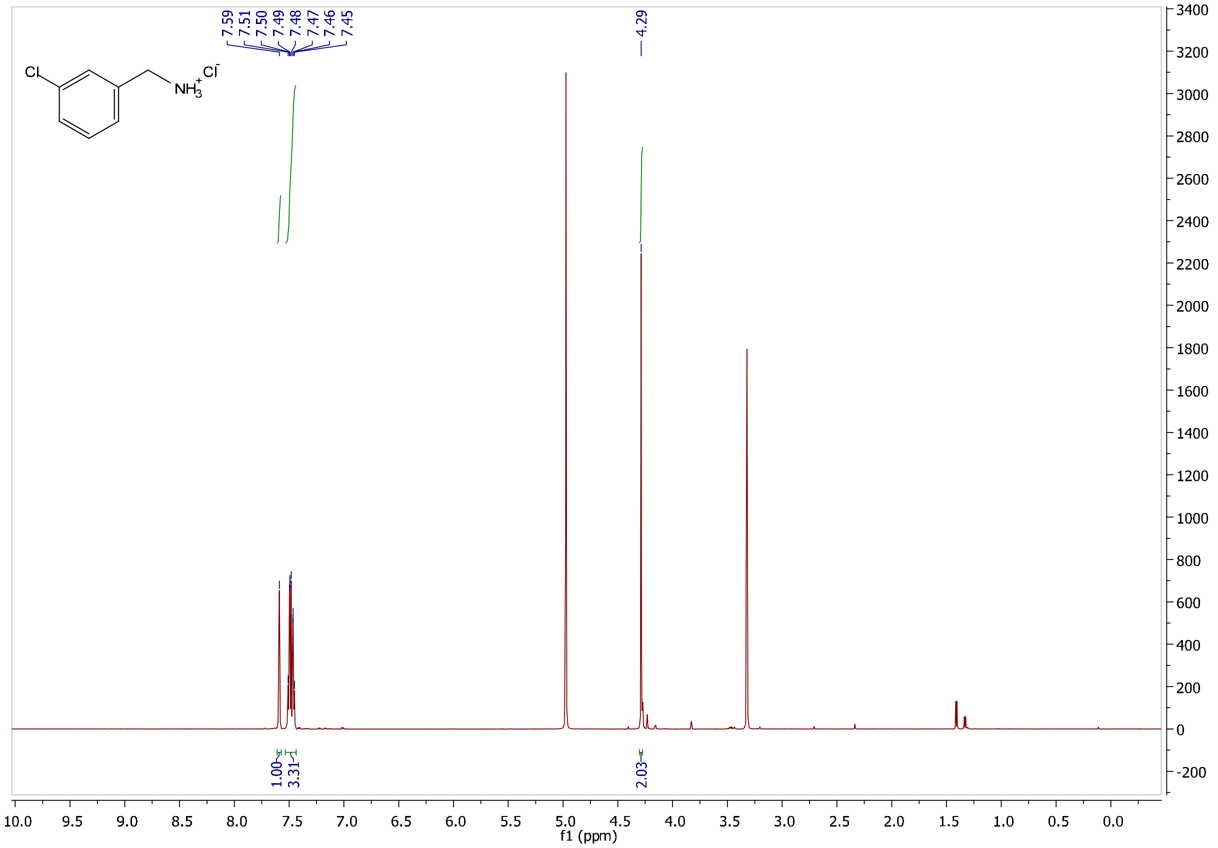

7.59
7.51
7.50
7.49
7.48
7.47
7.46
7.45
4.29
1.00
3.31
2.03
f1 (ppm)

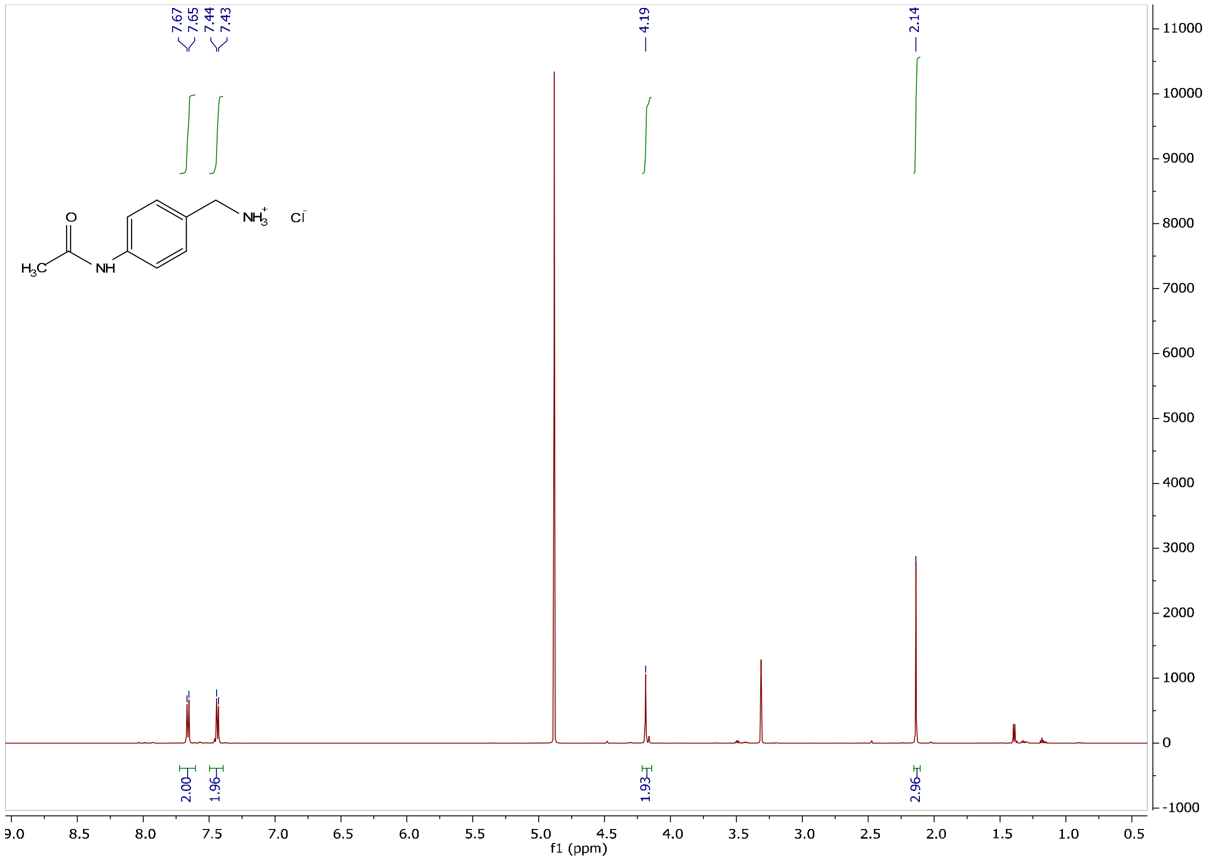

7.67
7.65
7.44
7.43
4.19
2.14
2.00
1.96
1.93
2.96
f1 (ppm)

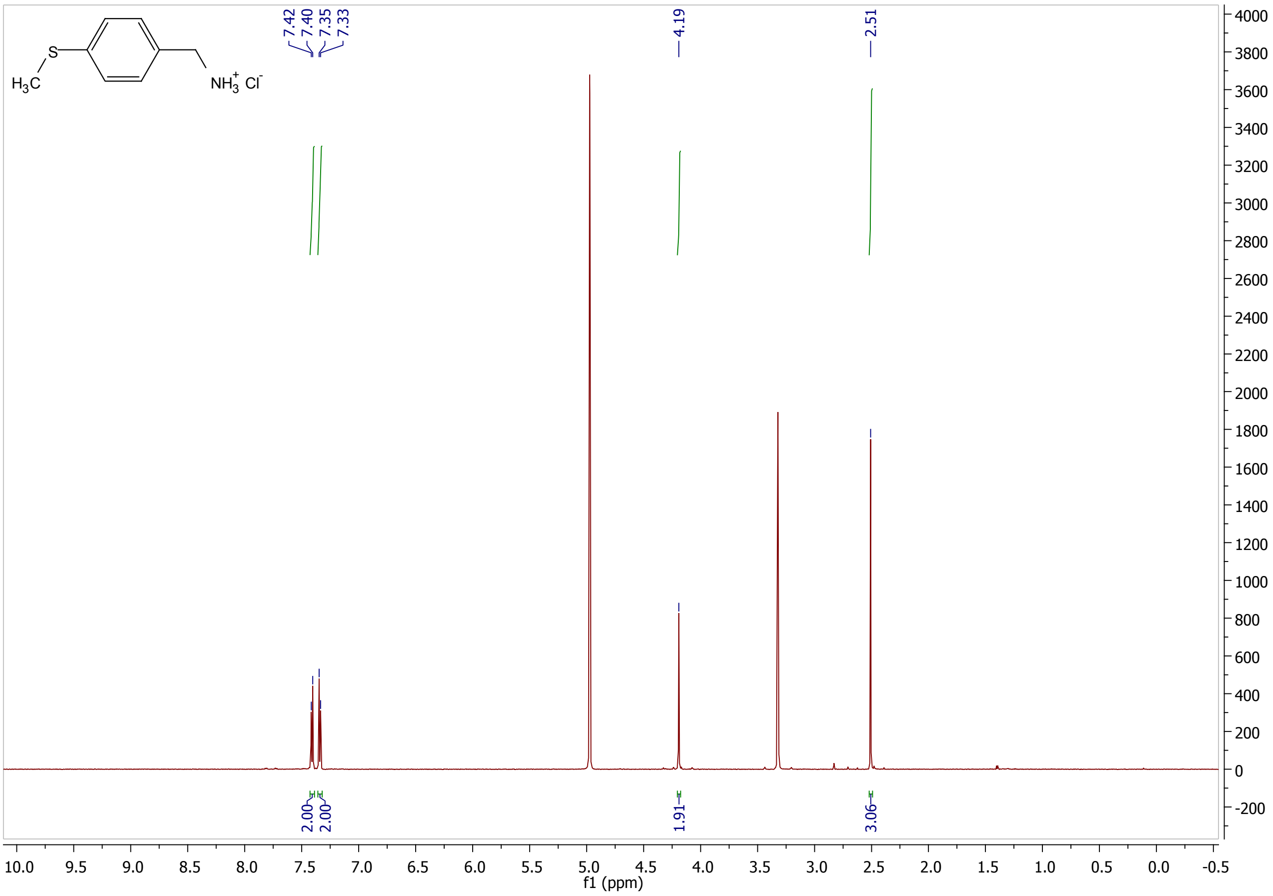

References

(1) ThalesNano. Phoenix Reactor http://thalesnano.com/phoenix-flow-reactor (accessed Jun 2, 2017).

(2) Adam, R.; Alberico, E.; Baumann, W.; Drexler, H.-J.; Jackstell, R.; Junge, H.; Beller, M. *Chem. – A Eur. J.* **2016**, *22*, 4991.

(3) Su, B.; Deng, M.; Wang, Q. *Adv. Synth. Catal.* **2014**, *356*, 977.

(4) Saito, Y.; Ishitani, H.; Ueno, M.; Kobayashi, S. *ChemistryOpen* **2017**, *6*, 211.

(5) Gandhamsetty, N.; Jeong, J.; Park, J.; Park, S.; Chang, S. *J. Org. Chem.* **2015**, *80*, 7281.

(6) Meade, E. A.; Sznaidman, M.; Pollard, G. T.; Beauchamp, L. M.; Howard, J. L. *Eur. J. Med. Chem.* **1998**, *33*, 363.
